# Different reactive profiles of calmodulin in the CSF samples of Chinese patients of four types of genetic prion diseases

**DOI:** 10.3389/fnmol.2024.1341886

**Published:** 2024-02-08

**Authors:** Xiao-Xi Jia, Chao Hu, Cao Chen, Li-Ping Gao, Dong-Lin Liang, Wei Zhou, Run-Dong Cao, Kang Xiao, Qi Shi, Xiao-Ping Dong

**Affiliations:** ^1^National Key-Laboratory of Intelligent Tracking and Forecasting for Infectious Disease, NHC Key Laboratory of Medical Virology and Viral Diseases, Collaborative Innovation Center for Diagnosis and Treatment of Infectious Diseases (Zhejiang University), National Institute for Viral Disease Control and Prevention, Chinese Center for Disease Control and Prevention, Beijing, China; ^2^Xuanwu Hospital Capital Medical University, Beijing, China; ^3^Center for Biosafety Mega-Science, Chinese Academy of Sciences, Wuhan, China; ^4^China Academy of Chinese Medical Sciences, Beijing, China; ^5^Shanghai Institute of Infectious Disease and Biosafety, Shanghai, China

**Keywords:** genetic prion diseases, cerebrospinal fluid, calmodulin, tau, 14-3-3

## Abstract

**Background and purpose:**

Calmodulin (CaM) levels exhibit significant elevation in the brain tissue of rodent and cell line models infected with prion, as well as in the cerebrospinal fluid (CSF) samples from patients diagnosed with sporadic Creutzfeldt-Jakob disease (sCJD). However, the status of CSF CaM in patients with genetic prion diseases (gPrDs) remains unclear. This study aims to assess the characteristics of CSF CaM in Chinese patients presenting four subtypes of gPrDs.

**Methods:**

A total of 103 CSF samples from patients diagnosed with T188K-gCJD, E200K-gCJD, D178N-FFI, P102L-GSS were included in this study, along with 40 CSF samples from patients with non-prion diseases (non-PrDs). The presence of CSF CaM and 14-3-3 proteins was assessed using Western blots analysis, while levels of CSF 14-3-3 and total tau were measured using enzyme-linked immunosorbent assays (ELISAs). Statistical methods including multivariate logistic regression were employed to evaluate the association between CSF CaM positivity and relevant clinical, laboratory, and genetic factors.

**Results:**

The positive rates of CSF CaM were significantly higher in cases of T188K-gCJD (77.1%), E200K-gCJD (86.0%), and P102-GSS (90.9%) compared to non-PrD cases (22.5%). In contrast, CSF CaM positivity was slightly elevated in D178N-FFI (34.3%). CSF CaM positivity was remarkably high in patients who tested positive for CSF 14-3-3 by Western blot and exhibited high levels of total tau (≥1400 pg/ml) as measures by ELISA. Multivariate logistic regression analysis confirmed a significant association between CSF CaM positivity and specific mutations in *PRNP*, as well as with CSF 14-3-3 positivity. Furthermore, the diagnostic performance of CaM surpassed that of 14-3-3 and tau when analyzing CSF samples from T188K-gCJD and E200K-gCJD patients.

**Conclusion:**

Western blot analysis reveals significant variations in the positivity of CSF CaM among the four genotypes of gPrD cases, demonstrating a positive correlation with 14-3-3 positivity and elevated tau levels in CSF.

## Introduction

Human prion diseases (PrDs) are classified etiologically into sporadic, inherited, and acquired forms. The most prevalent form is sporadic Creutzfeldt-Jakob Disease (sCJD), accounting for about 85% of human PrDs. Acquired forms included iatrogenic CJD (iCJD), variant CJD (vCJD), and Kuru (Prusiner, 1998; Chen and Dong, 2016). Inherited cases constitute approximately 10–15% of human PrDs and are closely associated with dozens of mutations in the *PRNP* gene, including genetic CJD (gCJD), fatal familial insomnia (FFI), and Gerstmann-Sträussler-Scheinker (GSS) syndrome (Shi et al., 2015b,^2021^). Over the past decade, a novel technique called real-time quaking-induced conversion (RT-QuIC) has been developed, significantly improving the diagnosis of human PrDs (Rhoads et al., 2020). However, there is still a pressing need to identify new biomarkers in cerebrospinal fluid, blood and other peripheral fluids for diagnosing human PrD.

Calmodulin (CaM) is a crucial protein involved in regulating calcium ion signaling and controlling multiple biological processes. Previous studies have demonstrated abnormal increases in calmodulin levels in brain tissues from prion infected rodent models and prion infected cell line (Chen et al., 2014; Zhang et al., 2017). Moreover, analysis has revealed a significantly higher positive CaM ratio on Western blot analysis of CSF samples from sCJD patients, indicating its diagnostic potential (Chen et al., 2021). Nevertheless, the role of CaM in the CSF of patients with genetic PrD (gPrD) remains poorly understood.

In this study, we enrolled CSF samples from 103 Chinese gPrD cases, including P102L-GSS, D178N-FFI, T188K-gCJD, and E200K-gCJD. CaM-specific Western blots revealed significantly higher proportions of CaM positivity in the T188K-gCJD (77.1%), E200K-gCJD (86.4%), and P102L-GSS (90.9%) groups compared to non-CJD cases (22.5%), with a slightly elevated rate observed in the D178N-FFI (34.3%) group. The presence of CSF CaM showed a positive correlation with the levels of CSF 14-3-3 and tau. Remarkably higher rates of CaM positivity were detected in individuals who tested positive for 14-3-3 by Western blotting and exhibited high total tau levels (≥1400 pg/ml) as measured by ELISA. Further analysis demonstrated significant associations between CSF CaM positivity and gPrD-specific mutations as well as 14-3-3 positivity.

## Materials and methods

### Patient

A total of 103 different gPrD patients were enrolled in this study, including 35 cases of T188K-gCJD, 22 cases of E200K-gCJD, 35 cases of D178N-FFI, 11 cases of P102L-GSS. The diagnoses of gPrD cases were conducted by the National Surveillance for CJD (CNS-CJD) at the China CDC according to the Diagnostic Criteria for CJD issued by the Chinese National Health Commission. All gPrDs cases in this study were verified through *PRNP* sequencing. Except for two D178N-FFI cases with postmortem neuropathology, brain autopsies were not performed on the remaining patients. Additionally, 40 other patients who clinically did not meet the diagnosis criteria for PrD were enrolled as non-CJD cases in this study. These non-CJD cases consisted of Alzheimer’s disease (AD; *n* = 7), autoimmune encephalitis (*n* = 5), cerebral infarction (*n* = 5), viral encephalitis (*n* = 4), Parkinson’s disease (*n* = 3), vascular dementia (*n* = 3), central nervous system lymphoma (*n* = 3), Lewy body dementia (*n* = 2), cerebral vasculopathy (*n* = 2), chronic meningoencephalitis (*n* = 1), epilepsy (*n* = 1), glioma (*n* = 1), encephalomyelitis (*n* = 1), leptomeningeal lymphomatosis/leukemia (*n* = 1), and mitochondrial encephalopathy (*n* = 1) (Chen et al., 2021). None of these non-CJD patients underwent neuropathological assays. The demographic, neurological, clinical examination and laboratory characteristics of the enrolled patients of gPrD and non-CJD were summarized in Supplementary Table 1.

### CSF samples

The CSF samples of these patients were obtained by the medical staff at local hospitals through routine lumbar punctures. All CSF specimens were devoid of any blood contamination. Subsequently, the collected CSF specimens were transferred to the central laboratory at China CDC, where they were centrifuged at 2000 rpm for 1 min, followed by aliquoting and stored at −80^°^C. The results of routine CSF biochemistry analysis of the enrolled patients were retrieved from the CNS-CJD information system in local hospitals, demonstrating normal cell count, glucose level, and total protein level. The use of these storage samples was approved by the Ethics Committee of the National Institute for Viral Disease Control and Prevention, China CDC, and personal information was limited to age, gender, clinical variables, and symptoms of PrDs.

### Western blot (WB)

A volume of 20 μl CSF from each case was subjected to 12% sodium dodecyl sulfate-polyacrylamide gel electrophoresis (SDS-PAGE). The fractionated proteins were transferred electronically onto a nitrocellulose membrane (Whatman, USA) using a semi-dry blotting system (Bio-Rad, Hercules, CA, USA). Following blocking, the membrane was incubated with a 1:1000 dilution of polyclonal antibody against 14-3-3 (Santa Cruz Biological, Dallas, TX, USA) at room temperature (RT) for 2 h, or with a 1:1000 dilution of anti-CaM monoclonal antibody (mAb) (Millipore, Billerica, MA, USA; 05–173) overnight at 4^°^C. Subsequently, the membranes were further incubated with a 1:2000 dilution of horseradish peroxidase-conjugated goat-derived anti-mouse antibody (Jackson ImmunoResearch Labs, West Grove, PA, USA; 115–035–003) at RT for an additional period of 2 h. Finally, the blots were developed using an enhanced chemiluminescence system (ECL; PerkinElmer, Waltham, MA, USA; NEL103E001EA) and the images were captured by the ChemiDoc™ XRS + System with Image Lab software (Bio-Rad, CA, USA). Quantification analysis was performed using Image J software (National Institutes of Health, Bethesda, MD, USA) (Hu et al., 2022).

### ELISA for CSF 14-3-3

CSF 14-3-3 was quantified using a commercial available double antibody sandwich ELISA kit (CY8082, CircuLex, Japan). Briefly, 2.5 μl of CSF sample was diluted (1:40) with the provided dilution buffer and transferred into a 96-well plate coated with 14-3-3 Gamma according to the manufacturer’s instructions. After incubating at RT for 60 min, the plates were thoroughly washed and incubated with the solution of 14-3-3 Gamma antibody at RT for another 60 min. Subsequently, HRP-conjugated antibody was added and incubated at RT for an additional 60 min reactions were developed with the addition of the solution of substrate for 15 min and terminated with the stop solution. Each reaction was measured automatically at 450 nm in an ELISA reader (PerkinElmer, United States). The values of CSF 14-3-3 were correlated to the external standard curve supplied by the manufacturer and measured in arbitrary unit (AU) per ml (Chen et al., 2019).

### ELISA for CSF total tau

The levels of total CSF tau were measured with a commercial ELISA kit (81572, Innotest hTau-Ag, Belgium). Briefly, 25 μl of CSF each patient was added to the ELISA wells of the ELISA plate and incubated overnight at RT. After washing, 100 μl of HRP-conjugated detection antibodies were added. The reaction was developed by adding 100 μl substrate working solution and incubating for 30 min in darkness. Absorbance at 450 nm was measured automatically using a microplate reader (Perkin Elmer, USA) after terminating the reaction with the addition of 2 M H_2_SO_4_. CSF tau concentrations were calculated based on a standard tau curve (Chen et al., 2019).

### WB normalization using positive control

A positive control was included in the WB using BV2 cell lines from the same batch. The BV2 cell line, treated consistently, served as a reference for assessing protein expression levels. Subsequently, the intensity of protein bands in other samples was normalized to the corresponding band intensity observed in the BV2 positive control.

### Statistical analysis

The data were processed using GraphPad Prism (GraphPad Software, USA) and SPSS 26.0 statistical software (SPSS, Chicago, IL, USA). Descriptive statistics were expressed as median (range) for continuous variables and as percentage for categorical variables. Categorical variables were compared using the chi-squared test, while the Mann–Whitney *U*-test was employed to analyze continuous variables after assessing normal distribution with the Shapiro–Wilk test. Logistic regression was utilized for multivariate analysis. Receiver operating characteristics (ROC) curves and area under the curve (AUC) values were used to assess the diagnostic performance of CaM, 14-3-3, and tau.

### Ethics statement

The human CSF samples utilized in this study were stored within the specimen bank located at the center laboratory of CNS-CJD. Approval for utilizing these archived storage samples was obtained from the Ethics Committee of the National Institute for Viral Disease Control and Prevention, China CDC.

## Results

### Demographical and clinical characteristics of the patients with gPrDs and non-PrD

As shown in Supplementary Table 1, the median of onset ages of the group comprising a total of 103 gPrD cases (ranging from 24 to 85 year) and 40 non-PrD cases (ranging from 18 to 80 year) were comparable, being 57 y. Notably, D178N-FFI and P102L-GSS exhibited significantly younger median onset ages compared to T188K- and E200K-gCJD. EEG examinations revealed periodic sharp wave complexes (PSWCs) in 24.4% (19/78) of gPrDs and 7.7% (3/39) of non-PrD cases, with E200K-gCJD patients displaying a notably higher incidence of PSWCs on EEG recordings. MRI abnormalities associated with sCJD, including symmetrical or asymmetrical cortical “ribbon” signs on diffusion-weighted imaging (DWI), high signal intensity in the caudate/putamen, or high signal intensity in the bilateral posterior tuberosity of the thalamus on proton density phase images, were reported in 60.2% (62/103) of gPrDs cases and 32.5% (13/40) of non-PrD cases. Positive CSF detection for the protein marker 14-3-3 was observed in 60.2% (62/103) of gPrD cases and 32.5% (13/40) of non-PrD cases, particularly prevalent among T188K-gCJD and E200K-gCJD cases. The median CSF tau levels for gPrD and non-PrD groups were measured at about 6611.17 pg/ml (48.25 to 59485.51) and 267.70 pg/ml (70.60 to 68993.76), respectively. D178N-FFI showed significantly lower CSF tau levels than other three subtypes of gPrDs. The polymorphisms of codon 129 and codon 219 in *PRNP* were comparable between the two groups, predominate consisting of M129M and E219E genotypes. Significantly more gPrD cases displayed myoclonus, visual or cerebellar disturbance, and akinetic mutism.

### Positive ratios of CSF CaM by western blot in various gPrD cases

**FIGURE 1:**
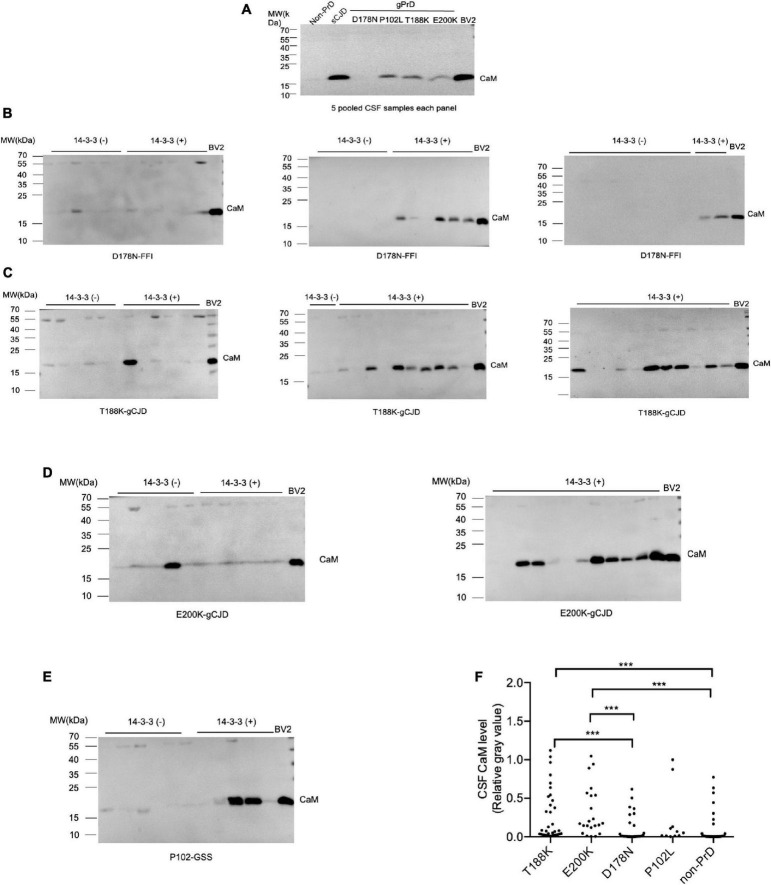
Determination of CaM in CSF specimens from the patients of gPrDs and non-PrD by Western blots. **(A)** Blots of various pooled CSF samples, including non-PrDs, sCJD, D178N-FFI, P102L-GSS, T188K-gCJD, E200K-gCJD. Each pooled sample consists of an equal amount of CSF from five individual patients. **(B)** Blots of 35 CSF specimens of D178N-FFI patients. **(C)** Blots of 35 CSF specimens of T188K-gCJD patients. **(D)** Blots of 22 CSF specimens of E200K-gCJD patients **(E)** Blots of 11 CSF specimens of P102L-GSS patients. The lysate of BV2 cells serves as a positive control and is utilized for the purpose of normalizing other samples. CaM-specific signals are marked with arrows on the right. The result of CSF 14-3-3 measured by Western blot on each CSF sample is presented above. **(F)** Quantitative assays of the relative gray value of the CaM signal of each tested CSF sample normalized to that of control. The *p*-values were described as ***p < 0.001, **p < 0.01, *p < 0.05, and ns, not significant.

To screen the CSF CaM in different types of PrDs, panels of pooled CSF specimens from 5 individual patients were prepared, including non-PrD, sCJD, T188K-gCJD, E200K-gCJD, D178N-FFI, and P102L-GSS. CaM-specific Western blots was performed using the lysate of BV2 cells as a positive control and for sample normalization. The results revealed a strong 17-kDa larger band in the pooled CSF samples of sCJD, T188K-gCJD, E200K-gCJD and P102L-GSS cases, whilst an extremely weak signal was observed in D178N-FFI cases and almost undetectable levels were found in non-CJD (Figure 1A). Subsequently, equal amounts of CSF specimens from gPrD and non-CJD cases were individually subjected to CaM-specific Western blots. Different intensities of specific CaM bands were observed among the various numbers of gPrDs cases (Figures 1B–E). The gray value of the CaM band in each case was screened and normalized with the respective CSF protein concentration. The relative gray values for CaM in the groups of T188K-gCJD and E200K-gCJD were significantly higher than D178N-FFI, P102L-GSS, and non-gPrD cases (Figure 1F).

**FIGURE 2:**
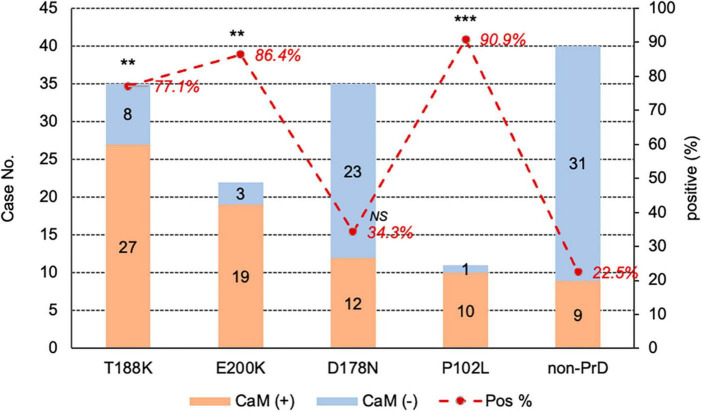
The positivity of CSF CaM in the groups of four different types of gPrD cases and non-PrD cases. The case numbers of CaM positive and negative in each group are shown in the column on right *Y*-axis and the positive percentage of CaM in each group is indicated at the **top** on the **left** *Y*-axis. The *p*-values were described as ***p < 0.001 and **p < 0.01.

Calculation of positive CaM cases revealed distinct profiles cross different disease groups (Figure 2). The positive ratios for P102L-GSS (90.9%, 10/11), E200K-gCJD (86.0%, 19/22), and T188K-gCJD (77.1%, 27/35) were higher than that for D178N-FFI (34.2%, 12/35) and non-CJD (22.5%, 9/40), showing statistical significances between disease groups (Table 1).

**TABLE 1:** Relative gray values and positivity of CSF CaM in four genotypes of gPrDs and non-PrDs.

Category	Disease	Mutation	No.	mean ± SD	*p*-value	Positive (%)	χ ^2^	*p*-value
gPrDs	gCJD	T188K^1^	35	0.2648 ± 0.3316	0.0425^a^	27 (77.1)	44.287	0.00001^b^
		E200K^1^	22	0.3232 ± 0.3214		19 (86.4)		
	FFI	D178N^2^	35	0.1544 ± 0.4190		12 (34.3)		
	GSS	P102L^1^	11	0.2086 ± 0.3640		10 (90.9)		
non-PrDs	non-PrDs	None^2^	40	0.0847 ± 0.1902		9 (22.5)		

CSF, cerebrospinal fluid; CaM, calmodulin; gPrDs, genetic prion diseases.

^a^One-way ANOVA test.

^b^Pearson chi-square test.

A significant difference between the groups marked with the different number (^1^ vs. ^2^) but no significant difference between the groups marked with the same number (^1^ vs.^1^, ^2^ vs. ^2^).

### Association of CSF CaM positivity with CSF 14-3-3 positivity by western blot and 14-3-3 levels by ELISA

The presence of CSF 14-3-3 of were determined in all enrolled cases using routine Western blot analysis. Among the total 103 gPrD cases, 60.2% (62/103) of tested positive, which was significantly higher compared to non-PrD cases (32.5%, 13/40). The positive rates of CSF 14-3-3 were 74.3% (26/35) in T188K-gCJD, 77.3% (17/22) in E200K-gCJD, 40.0% (14/35) in D178N-FFI, 45.5% (5/11) in P102L-GSS, respectively (Supplementary Table 1). Additionally, Table 2 demonstrates a higher prevalence of CaM positivity among CSF 14-3-3 positive gPrD cases compared to those that tested negative for CSF 14-3-3 (*p* = 0.003). Moreover, the CaM positivity in all four types of gPrD cases with 14-3-3 positivity was significantly higher than that in non-PrD groups, exhibiting statistical differences in T188K-gCJD, E200K-gCJD, and P102L-GSS but not in D178N-FFI (Table 2). These findings suggest a general association between CaM positivity and CSF 14-3-3 positivity by Western blotting across different genotypes of gPrDs.

**TABLE 2:** Relationship of CSF 14-3-3 positivity by Western blot and CSF CaM positivity in four genotypes of gPrDs and non-PrDs.

Disease	Mutation	CSF 14-3-3	CaM + (%)	CaM- (%)	*p*-value	OR (95% CI)	*p*-value [vs. 14-3-3 (+) in non-PrDs]
All gPrDs and non-PrDs		+	53 (70.7)	22 (29.3)	0.000^a^	4.417 (2.186–8.922)	N/A
		−	24 (35.3)	44 (64.7)			
All gPrDs		+	48 (77.4)	14 (22.6)	0.003^a^	3.600 (1.532–8.457)	0.014^c^
		−	20 (48.8)	21 (51.2)			/
gCJD	T188K	+	20 (76.9)	6 (23.1)	1.000^b^	0.952 (0.155–5.861)	0.033^b^
		−	7 (77.8)	2 (22.2)			/
	E200K	+	14 (82.4)	3 (17.6)	N/A	N/A	0.023^b^
		−	5 (100)	0 (0)			/
FFI	D178N	+	9 (64.3)	5 (35.7)	0.004^b^	10.800 (2.095–55.666)	0.257^b^
		−	3 (14.3)	18 (85.7)			/
GSS	P102L	+	5 (100.0)	0 (0.0)	N/A	N/A	0.036^b^
		−	5 (83.3)	1 (16.7)			/
non-PrDs		+	5 (38.5)	8 (61.5)	0.203^c^	3.594 (0.769–16.787)	N/A
		−	4 (14.8)	23 (85.2)			

CSF, cerebrospinal fluid; CaM, calmodulin; gPrDs, genetic prion diseases; gCJD, Creutzfeldt-Jacob disease; FFI, fatal familial insomnia; GSS, Gerstmann-Sträussler-Scheinker.

^a^Pearson chi-square test.

^b^Fisher exact test.

^c^Continuity-adjusted chi-square test.

N/A, not applicable.

/: Not within the study’s focus.

**FIGURE 3:**
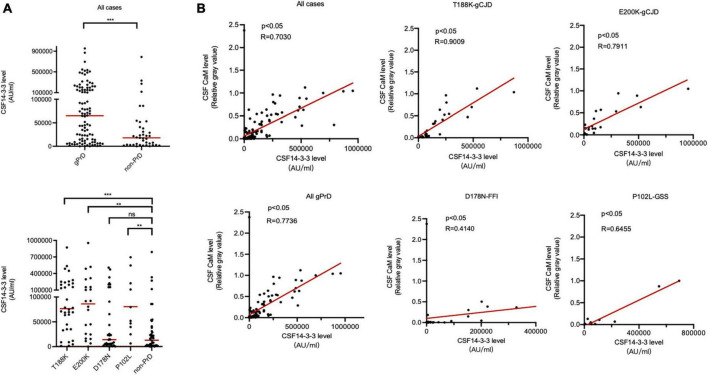
Association between CSF 14-3-3 levels by ELISA and CaM positivity by Western blot in the patients of four types of gPrD and non-PrD. **(A)** ELISA CSF 14-3-3 levels. Upper panel: CSF 14-3-3 levels of all included gPrD patients and non-PrDs. Lower panel: CSF 14-3-3 levels of four different types of gPrD cases and non-PrD patients. **(B)** The correlation between the levels of CSF 14-3-3 and the relative gray values of CaM. *Y*-axis: the relative grayscale values of CaM. *X*-axis: the levels of 14-3-3. The *p*-values were described as ***p < 0.001, **p < 0.01, *p < 0.05, and ns, not significant.

The CSF 14-3-3 levels of all cases were quantified using a commercial available ELISA kit. Remarkably, the median 14-3-3 level (57760.88 AU/ml) in gPrDs cases was significantly higher compared to non-PrD cases (18053.93 AU/ml) (Figure 3A, upper panel). In comparison to non-PrD cases, T188K-gCJD (76486.84 AU/ml), E200K-gCJD (86082.7 AU/ml), and P102L-GSS (65770.47 AU/ml) exhibited significantly elevated median CSF 14-3-3 levels, while D178N-FFI (14116.55 AU/ml) showed a slightly lower level without statistical difference (Supplementary Table 1 and Figure 3A, low panel). Statistical analysis demonstrated a strong linear correlation between CSF 14-3-3 levels and the relative gray value of CaM (Table 1) in total PrDs cases (*R* = 0.7736), as well as in T188K-gCJD (*R* = 0.9009), E200K-gCJD (*R* = 0.7911) and P102L-GSS (*R* = 0.6455) cases, however, this correlation was weak in D178N-FFI cases (*R* = 0.4140) (Figure 3B).

### Association of CSF CaM positivity with total CSF tau level by ELISA

Our previous study has identified a significant association of CSF CaM positivity with high levels (≥1400 pg/ml) of total CSF tau in patients with sCJD (Chen et al., 2021). The total tau levels in CSF specimens from all gPrD and non-CJD cases were measured by a commercial kit. Overall, 39.9% (57 out of 143 tested cases) showed high CSF tau levels (≥1400 pg/ml). Among them, 77.2% (44/57) of the cases with high CSF tau were CaM positive, while only 38.4% (33/86) of the cases with low CSF tau were positive, indicating a statistically significant difference (Table 3). The incidence rates of high CSF tau were 37.1% (13/35) in T188K-gCJD, 59.1% (13/22) in E200K-gCJD, 45.7% (16/35) in D178N-FFI, 54.5% (6/11) in P102L-GSS, and 22.5% (9/40) in non-PrD, respectively. Except for the group of E200K-gCJD, the other four groups showed relatively high proportions of CaM positivity among cases with high CSF tau, in which D178N-FFI and non-PrD revealed statistical differences (Table 3). Moreover, when comparing different gPrD cases with low CSF tau (<1400 pg/ml) to non-PrD cases also exhibiting low CSF tau, significantly higher ratios of CaM positivity were found in the group of T188K-gCJD, E200K-gCJD, and P102L-GSS, but not in that of D178N-FFI.

**TABLE 3:** Relationship of CSF tau level by ELISA and CSF CaM positivity in four genotypes of gPrDs and non-PrDs.

Disease	Mutation	CSF tau	CaM + (%)	CaM- (%)	Total (%)	*p*-value	OR (95% CI)	*p*-value (vs. <1400 pg/ml in non-PrDs)
All gPrDs and non-PrDs		<1400 pg/ml	33 (38.4)	53 (61.6)	86 (60.1)	0.000^a^	5.436 (2.552–11.578)	N/A
		≥1400 pg/ml	44 (77.2)	13 (22.8)	57 (39.9)			
All gPrDs		<1400 pg/ml	31 (56.4)	24 (43.6)	55 (53.4)	0.027^a^	2.604 (1.104–6.145)	0.00001^a^
		≥1400 pg/ml	37 (77.1)	11 (22.9)	48 (36.9)			/
gCJD	T188K	<1400 pg/ml	15 (68.2)	7 (31.8)	22 (62.9)	0.210^b^	5.600 (0.603–52.004)	0.00001^a^
		≥1400 pg/ml	12 (92.3)	1 (7.7)	13 (37.1)			/
	E200K	<1400 pg/ml	9 (100)	0 (0)	9 (40.9)	0.240^b^	N/A	0.00001^c^
		≥1400 pg/ml	10 (76.9)	3 (23.1)	13 (59.1)			/
FFI	D178N	<1400 pg/ml	3 (15.8)	16 (84.2)	19 (54.3)	0.030^b^	6.857 (1.412–33.289)	0.560^c^
		≥1400 pg/ml	9 (56.3)	7 (43.2)	16 (45.7)			/
GSS	P102L	<1400 pg/ml	4 (80.0)	1 (20.0)	5 (45.5)	N/A	N/A	0.001^b^
		≥1400 pg/ml	6 (100.0)	0 (0.0)	6 (54.5)			/
non-PrDs		<1400 pg/ml	2 (6.5)	29 (93.5)	31 (77.5)	0.000^c^	50.750 (6.051–425.633)	N/A
		≥1400 pg/ml	7 (77.8)	2 (22.2)	9 (22.5)			

CSF, cerebrospinal fluid; CaM, calmodulin; gPrDs, genetic prion diseases; gCJD, Creutzfeldt-Jacob disease; FFI, fatal familial insomnia; GSS, Gerstmann-Sträussler-Scheinker.

^a^Pearson chi-square test.

^b^Fisher exact test.

^c^Continuity-adjusted chi-square test.

N/A, not applicable.

/: Not within the study’s focus.

**FIGURE 4:**
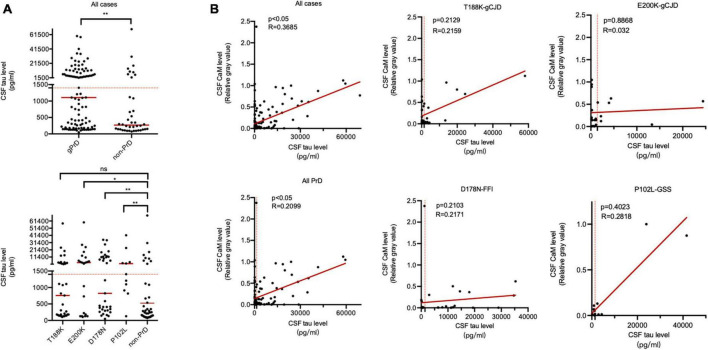
Association between CSF total tau levels by ELISA and CaM positivity by Western blot in the patients of four types of gPrD and non-PrD. **(A)** **Upper** panel: CSF total tau levels of all included patients of gPrD and non-PrD. **Lower** panel: CSF total tau levels of four different types of gPrD cases and non-PrD cases. **(B)** The correlation between the levels of total tau and the relative gray values of CaM. *Y*-axis: the relative grayscale values of CaM. *X*-axis: the levels of 14-3-3. The *p*-values were described as ***p < 0.001, **p < 0.01, *p < 0.05, and ns, not significant.

The median CSF tau values in total PrDs cases (1106.75 pg/ml) were higher than that of non-PrDs (267.70 pg/ml), among them, median CSF tau values of E200K-gCJD (3687.35 pg/ml) and P102L-GSS (1404.33 pg/ml) cases were notably high, whilst those of T188K-gCJD (756.66 pg/ml) and D178N-FFI (823.491 pg/ml) cases were slightly low without significance (Supplementary Table 1 and Figure 4A). Analysis of the linear correlation between the relative gray value of CaM and tau revealed a weak correlation in both total gPrD cases and the four subtypes of gPrDs (Figure 4B).

### Association of CSF CaM positivity with CSF 14-3-3 positivity by western blot and total CSF tau level by ELISA

All tested cases were classified into four groups according to the results of CSF 14-3-3 by Western blot and CSF tau by ELISA, 14-3-3 (+)/tau (≥1400 pg/ml), 14-3-3 (+)/tau (<1400 pg/ml), 14-3-3 (-)/tau (≥1400 pg/ml), 14-3-3 (-)/tau (<1400 pg/ml). The re activities of CSF CaM were evaluated and assigned to these four groups. As depicted in Table 4, the highest CaM positive ratio (86.5%) was observed in the group of 14-3-3 (+)/tau (≥1400 pg/ml), and the lowest ratio (25.0%) in that of 14-3-3 (-)/tau (<1400 pg/ml) in the context of all tested cases. A similar pattern was also observed in the context of gPrD cases, showing the highest CaM positive rate (84.4%) in the group of 14-3-3 (+)/tau (≥1400 pg/ml) and the lowest rate (40.0%) in that of 14-3-3 (-)/tau (<1400 pg/ml). The positive ratios of the groups of 14-3-3 (+)/tau (<1400 pg/ml) and 14-3-3 (-)/tau (≥1400 pg/ml) were comparable without statistical difference. These data highlight that the patients with CSF 14-3-3 positive and high CSF tau levels are highly likely to be positive for CSF CaM.

**TABLE 4:** Relationship of CSF 14-3-3 positivity by Western blot and CSF tau level with CSF CaM positivity.

Group	All gPrDs and non-PrDs			All gPrDs		
	**CaM + (%)**	**CaM- (%)**	***p*-value**	**CaM + (%)**	**CaM- (%)**	***p*-value**
14-3-3 (+)/tau > 1400 pg/ml	32 (86.5)^1^	5 (13.5)	0.00001*	27 (84.4)^1^	5 (15.6)	0.005*
14-3-3 (+)/tau < 1400 pg/ml	21 (55.3)^2^	17 (44.7)		21 (70.0)^1,2^	9 (30)	
14-3-3 (-)/tau > 1400 pg/ml	12 (60.0)^1,2^	8 (40)		10 (62.5)^1,2^	6 (37.5)	
14-3-3 (-)/tau < 1400 pg/ml	12 (25.0)^3^	36 (75)		10 (40.0)^2^	15 (60)	

CSF, cerebrospinal fluid; CaM, calmodulin; gPrDs, genetic prion diseases.

A significant difference between the groups marked with the different number (^1^ vs. ^2^, ^1^ vs. ^3^, ^1^ vs. ^2^,^1.2^ vs. ^3^) but no significant difference between the groups marked with the same number(s) (^1^ vs.^ 1^, ^2^ vs. ^2^,^1,2^ vs.^ 1^, ^1,2^ vs. ^2^).

*Pearson chi-square test.

### Association of CSF CaM positivity with the main clinical features and clinical examinations of gPrDs

The numbers of CaM-positive and CaM-negative cases were separately determined in total gPrDs and four individual types of gPrDs based on the main clinical elements and clinical examinations, including gender, onset ages, clinical durations, MRI abnormalities, PSWC on EEG, as well as dementia and other neurological manifestations (Table 5). Statistical analysis revealed significantly differences only in the clinical duration among total gPrDs (*p* = 0.033) and in T188K-gCJD (*p* = 0.047). Other items, regardless of total gPrDs or the special genotypes of gPrDs, did not reveal significance.

**TABLE 5:** Relationship of clinical features and CFS CaM positivity in total and four genotypes of gPrDs.

Clinical	All gPrDs			T188K-gCJD			E200K-gCJD			D178N-FFI			P102L-GSS		
	**CaM +** **(*n* = 68)**	**CaM-** **(*n* = 35)**	***p*-value**	**CaM +** **(*n* = 27)**	**CaM-** **(*n* = 8)**	***p*-value**	**CaM +** **(*n* = 19)**	**CaM-** **(*n* = 3)**	***p*-value**	**CaM +** **(*n* = 12)**	**CaM-** **(*n* = 23)**	***p*-value**	**CaM +** **(*n* = 10)**	**CaM-** **(*n* = 1)**	***p*-value**
Gender (M/F)	34/34	16/19	0.680^a^	16/11	5/3	0.869^b^	6/13	2/1	0.527^b^	7/5	9/14	0.311^b^	5/5	0/1	N/A
Median onset age (y) (range)	57.5 (34, 85)	53 (24, 76)	0.157^c^	62 (40,85)	60.5 (50, 76)	0.969^c^	58 (42, 70)	50 (44, 63)	0.416^c^	56 (42, 61)	50 (24, 70)	0.394^c^	48.5 (34,67)	63 (63,63)	0.205^c^
Median disease duration (m) (min, max)	8 (4, 19)	5 (2, 25)	0.033^c^	4 (2,12)	9.5 (4, 13)	0.047^c^	5 (4, 5)	19 (19,19)	0.114^c^	14 (8,25)	7.5 (4,13)	0.067^c^	8 (4,12)	N/A	N/A
MRI abnormality no. (%)	39 (57.4)	23 (65.7)	0.412^a^	6 (22.2)	0 (0.0)	0.299^b^	16 (84.2)	3 (100.0)	N/A	9 (75.0)	19 (82.6)	0.670^b^	8 (80.0)	1 (100.0)	N/A
PSWC in EEG no. (%)	16 (29.1)	3 (13.0)	0.132^a^	5 (19.2)	2 (28.6)	0.623^b^	10 (66.7)	1 (100.0)	N/A	0 (0.0)	0 (0.0)	/	1 (10.0)	1 (0.0)	N/A
Progressive dementia no. (%)	60 (88.2)	31 (88.6)	0.960^d^	24 (88.9)	5 (62.5)	0.117^b^	16 (84.2)	3 (100.0)	1.000^b^	11 (91.7)	22 (95.7)	1.000^b^	9 (90.0)	1 (100.0)	N/A
Myoclonus no. (%)	42 (61.8)	22 (62.9)	0.914^a^	16 (59.3)	4 (50)	0.700^b^	13 (68.4)	1 (33.3)	0.527^b^	8 (66.7)	17 (73.9)	0.706^b^	5 (50.0)	0 (0.0)	N/A
Visual or cerebellar disturbance no. (%)	42 (61.8)	22 (62.9)	0.914^a^	13 (48.1)	3 (37.5)	0.700^b^	17 (89.5)	2 (66.7)	N/A	6 (50.0)	17 (73.9)	0.261^b^	6 (60.0)	0 (0.0)	N/A
Pyramidal or extrapyramidal dysfunction no. (%)	53 (77.9)	26 (74.3)	0.678^a^	20 (74.1)	3 (37.5)	0.091^b^	16 (84.2)	2 (66.7)	N/A	10 (83.3)	20 (87.0)	1.000^b^	7 (70.0)	1 (100.0)	N/A
Akinetic Mutism no. (%)	24 (35.8)	19 (54.3)	0.073^a^	6 (22.2)	1 (12.5)	1.000^b^	9 (52.6)	2 (33.3)	1.000^b^	6 (50.0)	16 (69.6)	0.292^b^	3 (30.0)	0 (0.0)	N/A

CSF, cerebrospinal fluid; CaM, calmodulin; gPrDs, genetic prion diseases; MRI, magnetic resonance imaging; PWSC, periodic sharp wave complexes; EEG, electroencephalogram.

^a^Pearson chi-square test.

^b^Fisher exact test.

^c^Mann-Whitney *U*-test.

^d^Continuity-adjusted chi-square test.

N/A, not applicable.

Subsequently, logistic regression analysis was performed to assess the impact of multiple variables on CSF CaM positivity by Western blot. As shown in Supplementary Table 2, T188K (*p* = 0.009), E200K (*p* = 0.001), and CSF 14-3-3 (*p* = 0.000) showed significantly correlation with CaM positivity, whereas CSF tau level was not.

### Evaluation of the diagnostic performance of CSF CaM in four genotypes of gPrDs

**FIGURE 5:**
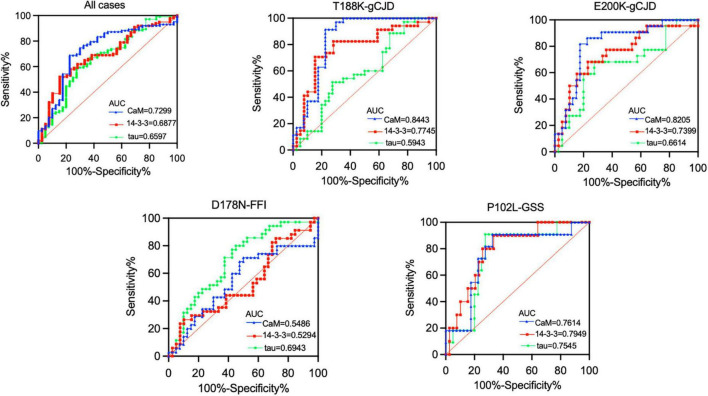
Receiver operating characteristics (ROC) curve analysis in comparison of the performance of three CSF biomarkers (CaM, 14-3-3, and tau) across four genotypes of gPrDs. ROC curves illustrate the sensitivity and specificity of various CSF biomarker combinations in the differential diagnosis in four genotypes of gPrDs. AUC values are listed with blue (CaM), red (14-3-3), and green (tau) lines on the lower right of each picture.

The diagnostic accuracy of CaM, 14-3-3, and tau protein in distinguishing between gPrD and non-PrD patients was assessed by calculating the area under the ROC curve (Figure 5). The results revealed that CaM had superior diagnostic performance across all cases (AUC = 0.7299), particularly for T188K-gCJD (AUC = 0.8443) and E200K-gCJD (AUC = 0.8205) cases. For the diagnosis of P102L-GSS, there was minimal disparity in the performance of the three biomarkers. However, it is worth nothing that both CaM and 14-3-3 had AUC values close to 0.5 for diagnosing D178N, indicating inferior diagnostic accuracy compared to tau protein (AUC = 0.6943).

## Discussion

In this study, we have identified that similar as the data of previously reported sCJD cases (Chen et al., 2021), high ratios of Chinese patients with T188K-gCJD, E200K-gCJD, and P102L-GSS exhibit positive CaM results in their CSF specimens by Western blot in the period of disease. On the contrary, the positive rate of CaM in CSF samples from patients with D178N-FFI is remarkably lower than that observed in these three subtypes of gPrDs and even comparable to non-CJD cases. These findings suggest that positive CaM in CSF by Western blot is not only frequent in sCJD cases but also present in some types of gPrD cases, thereby potentially serving as a valuable biomarker for diagnosing human PrDs.

Two distinct phenotypes of CSF CaM reactivity in Western blot were observed among the four types of gPrDs enrolled in this study, with high positive rates found in the groups of gCJD and GSS, while a low positive rate was observed in that of FFI. It is well known that over 55 different types of mutations in the *PRNP* gene are directly associated with human gPrDs worldwide (Jeong and Kim, 2014). The specific type and position of *PRNP* mutations, as well as polymorphism at certain positions (e.g., codons 129 and 219), largely determine the phenotypes of disease. Three major subtypes of human gPrDs, namely gCJD, GSS, and FFI, not only display different clinical phenotypes but also exhibit different neuropathological abnormalities and PrP^Sc^ molecular features (Zerr and Poser, 2002). Apparently, there appears to be no association between *PRNP* variants. In China, T188K-gCJD, D178N-FFI, and E200K-gCJD are the most common forms of gPrDs (Shi et al., 2008, 2012, 2015b; 2021; 2022; Chen and Dong, 2016; Gao et al., 2019), which differ from those seen among Caucasian or even neighboring countries such as Japan and Republic of Korea (Choi et al., 2009; Hamaguchi, 2013; Lee et al., 2014). Our previous studies have addressed similarities between Chinese T188K-gCJD and E200K-gCJD patients based on clinical examinations, including CSF 14-3-3 positivity, these findings are also similar to those seen among sCJD patients (Chen et al., 2013; Gao et al., 2019). Contrarily, D178N-FFI exhibits different clinical, neuropathological, and PrP^Sc^ pathogenic processes compared to sCJD and the majority of gCJDs (Chen et al., 2018). The phenotypic difference between FFI and other gPrD patients may be attributed to variations in the conversion rate of PrP^C^ to PrP^Sc^, resulting in a higher deposition of PrP^Sc^ observed in the neuropathology of other gPrD patients than FFI. This discrepancy directly impacts the extent of neuronal damage across various brain regions, leading to significantly lower positive ratios and the levels of CSF 14-3-3 and total tau in FFI cases as compared to sCJD as well as T188K-gCJD and E200K-gCJDs. Despite different in clinical presentation and neuropathology between GSS with sCJD and gCJD, elevated levels of CSF 14-3-3 and tau are observed in P102L-GSS cases. It seems that frequent CSF CaM positivity on Western blot is observed among gPrDs that resemble sCJD.

Although abnormal alterations of CaM have been observed in the brains of the patients with neurodegenerative diseases and animal models, including prion disease (O’Day and Myre, 2004; Bartolome et al., 2007; Esteras et al., 2012), the exact mechanism underlying the presence of positive CaM in CSF samples from PrDs remains unclear. Our previous proteomic analysis of global proteins in brains of human PrDs has demonstrated upregulated CaM levels in the cortex regions of sCJD (Shi et al., 2015a). Another proteomic study analyzing CSF samples from sCJD patients found that CaM is the 3^rd^ highest up-regulated protein compared to non-PrD patients (Chen et al., 2014). Significant elevation of CaM has also been detected in the brains of prion-infected rodents and sCJD patients (Zhang et al., 2017), suggesting a potential link between elevated CaM levels in both brain tissue and CSF samples from human PrDs. However, changes in other proteins within the brain and CSF do not show consistent pattern. Protein 14-3-3 is frequently detected at high levels in CSF specimens from sCJD patients, while brain 14-3-3 levels seem to be minimally altered in sCJD patients or even downregulated in scrapie-infected experimental hamsters (Shi et al., 2014). Total tau levels are markedly increased in sCJD cases, but the alterations to tau within the brains of human PrDs are more complex, characterized by disturbances of tau phosphorylation (Skillback et al., 2014). Despite extensive dysregulation of CSF proteins observed during the diseased phase of human PrDs, it is evident that distinct dysfunction pathways exist, which may even operate independently for individual proteins.

Similar to the changes observed in 14-3-3 and tau proteins in CSF, the presence of CaM in CSF is not specific to prion disease. Positive detection of CaM in CSF can also be found in a small subset of non-PrD cases. Although certain clinical features, such as shorter disease duration observed in T188K-gCJD cases here, as well as PSWC on EEG, abnormality MRI findings, and sampling time in sCJD cases previously reported (Chen et al., 2013, 2021) show some correlation with CSF CaM positivity, further assays of multivariate logistic regression failed to figure out statistical significance. Two factors, CSF 14-3-3 and CSF total tau levels, demonstrate are more meaningful associations. The significances of CSF CaM positivity with CSF total tau has been addressed for sCJD patients (Chen et al., 2021), while its association with CSF 14-3-3 has been explored for gPrD patients through univariate analysis. However, it is important to acknowledge that both our previous study on sCJD (Chen et al., 2021) and this current study on gPrDs had relatively limited sample sizes. Therefore, a more comprehensive investigation using larger-scale samples is necessary to identify correlative factors for CaM positivity in human PrDs. Additionally, while this study strictly adhered to inclusion criteria for the control group diagnosis based solely on clinical assessment, accurate determining control case diagnoses remains challenging due to the absence of neuropathological confirmation. Consequently, this study primarily emphasizes the specificity of CSF CaM detection within different genotypes of gPrDs and its differential distribution compared to other neurodegenerative diseases, detailed comparative analyses will be conducted in future investigations.

Recently, the repeated validation of RT-QuIC based on CSF and skin specimens has demonstrated its significance for sCJD (Xiao et al., 2020, 2021; Mastrangelo et al., 2023). However, the application of RT-QuIC for various types of gPrDs is still pending. Our previous study involving five types of gPrDs cases revealed a relatively lower sensitivity of CSF RT-QuIC compared to sCJD (Shi et al., 2021), thus necessitating alternative diagnostic methodologies. One potential solution lies in implementing a combined testing scheme, as higher rates of CSF CaM positivity have been observed in patients with both CSF 14-3-3 positivity and elevated CSF tau level in previous and current studies. This approach holds promise for enhancing diagnostic specificity not only for sCJD but also certain types of gPrDs.

## Data availability statement

The original contributions presented in the study are included in the article/Supplementary material, further inquiries can be directed to the corresponding authors.

## Ethics statement

The studies involving humans were approved by the National Institute for Viral Disease Control and Prevention, China CDC. The studies were conducted in accordance with the local legislation and institutional requirements. The participants provided their written informed consent to participate in this study. The animal study was approved by National Institute for Viral Disease Control and Prevention, China CDC. The study was conducted in accordance with the local legislation and institutional requirements.

## Author contributions

X-XJ: Conceptualization, Data curation, Formal Analysis, Methodology, Visualization, Writing – original draft. CH: Conceptualization, Formal Analysis, Methodology, Visualization, Writing – original draft. CC: Funding acquisition, Supervision, Writing – review and editing. L-PG: Funding acquisition, Writing – original draft. D-LL: Methodology, Formal Analysis, Writing – original draft. WZ: Project administration, Resources, Writing – original draft. R-DC: Methodology, Writing – original draft. KX: Methodology, Writing – original draft. QS: Resources, Supervision, Writing – review and editing. X-PD: Resources, Supervision, Writing – review and editing.
