# Supplementary material for: Different reactive profiles of calmodulin in the CSF samples of Chinese patients of four types of genetic prion diseases
Source: Front Mol Neurosci. 2024 Feb 8;17:1341886. doi: 10.3389/fnmol.2024.1341886 (PMC10881788; doi:10.3389/fnmol.2024.1341886)
Supplement: Supplementary file 1 [file Table_1.DOCX]

Table S1. The demography and clinical characteristics of the patients with gPrDs and non-PrDs

| Clinical features | Total gPrD (n=103) | | T188K-gCJD (n=35) | | E200K-gCJD (n=22) | | D178N-FFI (n=35) | | P102L-GSS (n=11) | | non-PrD (n=40) | |
| --- | --- | --- | --- | --- | --- | --- | --- | --- | --- | --- | --- | --- |
|  |  | p -value *vs.* non-PrD |  | p -value *vs.* non-PrD |  | p -value *vs.* non-PrD |  | p -value *vs.* non-PrD |  | p -value *vs.* non-PrD |  | p -value *vs.* Four mutations |
| Gender (M/F) | 50/53 | 0.336^b^ | 21/14 | 0.826^b^ | 8/14 | 0.111^b^ | 16/19 | 0.308^b^ | 5/6 | 0.712^c^ | 23/17 | 0.377^b^ |
| Median age at onset (range) (y) | 57 (24-85) | 0.617^a^ | 62 (40,85) | 0.144^a^ | 57 (42,70) | 0.837^a^ | 53 (24,70) | 0.062^a^ | 49 (34,67) | 0.176^a^ | 57 (18-80) | 0.004^e^ |
| Age at onset <50 years no. (%) | 28/103 (27.2) | 0.736^b^ | 3/35 (12) | 0.021^b^ | 5/22 (22.7) | 0.539^b^ | 14/35 (40) | 0.364^b^ | 6/11(54.5) | 0.249^c^ | 12/40 (30) | 0.008^d^ |
| Age at onset 50-70 years no. (%) | 69/103 (67) | 0.107^b^ | 26/35 (74.3) | 0.052^b^ | 17/22 (77.3) | 0.055^b^ | 21/35 (60) | 0.514^b^ | 5/11 (45.5) | 0.679^b^ | 21/40 (52.5) | 0.008^d^ |
| Age at onset >70 years no. (%) | 6/103 (5.8) | 0.063^c^ | 6/35 (17.1) | 0.967^b^ | 0/22 (0) | 0.096^c^ | 0/35 (0) | 0.028^c^ | 0/11 (0) | 0.318^c^ | 7/40 (17.5) | N/A |
| Codon 129 genotype Met-Met/Total (%) | 103/103 (100) | N/A | 35/35（100） | N/A | 22/22（100） | N/A | 35/35（100） | N/A | 11/11（100） | N/A | 38/38 (100) | N/A |
| Codon 219 genotype Glu-Glu/Total (%) | 73/74 (98.6) | 1^b^ | 24/25 (96) | 1^d^ | 19/19 (100) | 1^d^ | 22/22 (100) | 1^d^ | 8/8 (100) | 1^d^ | 37/38 (97.4) | N/A |
| PSWC in EEG (%) | 19/78 (24.4） | 0.030^b^ | 7/33 (21.2) | 0.273^c^ | 11/16 (68.8) | 0.001^b^ | 0/25 (0) | 0.449^c^ | 1/4 (25) | N/A | 3/39 (7.7） | 0.100^d^ |
| MRI abnormal change/Total no. (%) | 62/103 (60.2) | 0.003^b^ | 6/35 (17.1) | 0.127^b^ | 19/22 (86.4) | 0.00001^b^ | 28/35 (80) | 0.00001^b^ | 9/11 (81.8) | 0.010^c^ | 13/40 (32.5) | 0.00001^b^ |
| CSF 14-3-3 Positive/Total no. (%) | 62/103 (60.2) | 0.003^b^ | 26/35 (74.3) | 0.000^b^ | 17/22 (77.3) | 0.001^b^ | 14/35(40) | 0.500^b^ | 5/11 (45.5) | 0.660^c^ | 13/40 (32.5) | 0.008^d^ |
| CSF 14-3-3 median value (AU/ml, min, max) | 57760.87 (27.38,953644.59) | 0.004^a^ | 76486.83 (1427.73, 871200.81) | 0.00001^a^ | 86082.69 (27.38, 953644.59) | 0.001^a^ | 14116.55 (735.25, 507378.201) | 0.359^a^ | 65770.46 (6472.37, 693783.19) | 0.004^a^ | 18053.93 (121.35,789107.14) | 0.00001^e^ |
| CSF total tau median value (pg/ml, min, max) | 1106.75 (48.25,59485.51) | 0.003^a^ | 756.65 (105.17，58054.78) | 0.161^a^ | 3687.35 (105.17, 59485.50) | 0.037^a^ | 823.49 (48.25, 35318.83) | 0.004^a^ | 1404.33 (126.77, 41589.99) | 0.010^a^ | 267.70  (70.60,68993.76) | 0.014^e^ |
| Progressive dementia/Total no. (%) | 91/103 (88.3) | 0.196^b^ | 29/35 (82.9) | 0.751^b^ | 19/22 (86.4) | 0.779^c^ | 33/35 (94.3) | 0.140^c^ | 10/11 (91) | 0.694^c^ | 32/40 (80) | N/A |
| Myoclonus no. (%) | 64/103 (62.1) | 0.00001^b^ | 20/35 (57.1) | 0.001^b^ | 14/22 (63.6) | 0.001^b^ | 25/35 (71.4) | 0.00001^b^ | 5/11 (45.5) | 0.185^c^ | 8/40 (20) | 0.00001^d^ |
| Visual or cerebellar disturbance no. (%) | 64/103 (62.1) | 0.00001^b^ | 16/35 (45.7) | 0.004^b^ | 19/22 (86.4) | 0.00001^b^ | 23/35 (65.7) | 0.00001^b^ | 6/11 (54.5) | 0.019^c^ | 6/40 (15) | 0.00001^b^ |
| Pyramidal or extrapyramidal dysfunction no. (%) | 79/103 (76.7) | 0.407^b^ | 23/35 (71.4) | 0.691^b^ | 18/22 (81.8) | 0.309^b^ | 30/35 (85.7) | 0.105^b^ | 8/11 (72.7) | 1.000^c^ | 28/40 (70) | 0.304^c^ |
| Akinetic Mutism no. (%) | 43/103 (41.7) | 0.00001^b^ | 7/35 (20) | 0.101^c^ | 11/22 (50) | 0.00001^c^ | 22/35 (62.9) | 0.00001^b^ | 3/11 (27.3) | 0.104^c^ | 2/40 (5) | 0.00001^c^ |

Abbreviations: gPrDs, genetic prion diseases; PWSC, periodic sharp wave complexes; EEG, electroencephalogram; MRI, magnetic resonance imaging; CSF, cerebrospinal fluid. ^a^ Mann-Whitney *U* test

^b^ Pearson chi-square test

^c^ continuity-adjusted chi-square test

^d^ Fisher exact test

^e^ Kruskal-Wallis test

N/A: Not applicable

Table S2. Multivariate logistic regression of levels of CSF CaM and the related influence factors

| Factors | | p-value |
| --- | --- | --- |
| *PRNP* mutations | T188K | 0.009 |
|  | E200K | 0.001 |
|  | D178N | 0.821 |
|  | P102L | 0.998 |
| CSF test | CSF 14-3-3 | 0.00001 |
|  | CSF total tau | 0.249 |
